# Group size and visitor numbers predict faecal glucocorticoid concentrations in zoo meerkats

**DOI:** 10.1098/rsos.161017

**Published:** 2017-04-19

**Authors:** Katy Scott, Michael Heistermann, Michael A. Cant, Emma I. K. Vitikainen

**Affiliations:** 1 Centre for Ecology and Conservation, University of Exeter, Penryn, Cornwall TR10 9FE, UK; 2 German Primate Centre, Leibniz Institute for Primate Research, Göttingen, Germany

**Keywords:** faecal glucocorticoids, group size, animal welfare, zoo visitors, meerkat, *Suricata suricatta*

## Abstract

Measures of physiological stress in zoo animals can give important insights into how they are affected by aspects of their captive environment. We analysed the factors influencing variation in glucocorticoid metabolites in faeces (fGCs) from zoo meerkats as a proxy for blood cortisol concentration, high levels of which are associated with a stress response. Levels of fGCs in captive meerkats declined with increasing group size. In the wild, very small groups of meerkats are at a higher risk of predation, while in larger groups, there is increased competition for resources. Indeed, group sizes in captivity resemble those seen in unstable coalitions in the wild, which may represent a stressful condition and predispose meerkats to chronic stress, even in the absence of natural predators. Individuals in large enclosures showed lower levels of stress, but meerkat density had no effect on the stress measures. In contrast with data from wild meerkats, neither sex, age nor dominance status predicted stress levels, which may reflect less food stress owing to more equal access to resources in captivity versus wild. The median number of visitors at the enclosure was positively correlated with fGC concentrations on the following day, with variation in the visitor numbers having the opposite effect. Our results are consistent with the hypothesis that there is an optimum group size which minimizes physiological stress in meerkats, and that zoo meerkats at most risk of physiological stress are those kept in small groups and small enclosures and are exposed to consistently high numbers of visitors.

## 1. Introduction

Measuring stress in zoo animals is important to improve welfare and monitor the effect of captivity, but is difficult in practice. Variation between species and between individual animals in their behavioural responses to a stressor make it difficult to define fixed, reliable criteria for assessing animal welfare based on their rearing conditions [1,2]. Observational measures, such as behavioural repertoire, or breeding success can provide useful information [3]. For example, large numbers of visitors may be stressful to the animals and linked with changes in their behaviour [4]. Yet, they may also be difficult to interpret and to causally link to a particular set of conditions the animals are experiencing, for example, because the behaviour of the animals could be the driver of variation in visitor numbers, as well as the consequence of it [5,6]. A useful method, therefore, is to measure directly how variation in captive conditions and exposure to visitors impacts the physiological response of the animals, by measuring components of the hormonal stress response [7].

The main characteristic of the physiological stress response in vertebrates is the release of glucocorticoids (cortisol, corticosterone) from the adrenal gland in response to a stressor. Glucocorticoids play an essential role in general homeostasis, and their presence at elevated concentrations can also indicate a stress response, as one of their functions is to trigger the mobilization of energy stores to allow the animal to respond to the current threat [1,8,9]. Analysing the level of glucocorticoids in the animal's bloodstream is a way to measure the level of the hormonal stress response at a given time. However, this requires catching the animal and extracting a blood sample: this is impractical in zoos, and capture itself constitutes a stressor which will compromise future samples for as long as the stress response lasts, and possibly even longer if a stressor results in longer term changes in an animal's state [7,9]. An alternative method is to analyse the level of glucocorticoid metabolites in excreta, for example, a faecal sample from the animal (faecal glucocorticoids or faecal glucocorticoid metabolites (fGCs)). The amount of fGCs provides an estimate for glucorticoid production over the preceding hours or days, depending on the rate of metabolism and volume of throughput [7]. For the same animal, or conspecifics on similar diets, this provides a relative measure of hormonal stress response at different times or in different situations. Using faecal sampling to monitor glucocorticoid levels in both captive-housed animals and those living in the wild has become a widespread technique over recent years [7,10].

The key question when studying the glucocorticoid response of zoo animals is how characteristics of the captive environment, social group and those of the individual itself affect its stress response. Quantifying stress is not straightforward, however, as there are no clear guidelines of what constitutes an ‘elevated’ response, and as aspects of captivity may in several ways be masking the stress response of the animals. Comparisons to wild conspecifics are limited, in that scarcity of food is a major source of stress for animals living in the wild, yet often completely absent in animals in captivity that are typically fed to requirement. Furthermore, chronic stress in captive animals may lead to downregulation or suppression of the stress response (hypothalamic-pituitary-adrenal axis; [11]), leading to highly stressed animals scoring misleadingly low in measures of physiological stress. Therefore, individual variation in stress response measured against a range of conditions, if not the absolute levels of the stress hormones, may better indicate how aspects of the environment affect the animals' experience. Keeping these limitations in mind, comparisons to wild conspecifics may be a useful method to understand how the conditions experienced by captive animals are affecting their cortisol levels, and to guide decisions on how to best minimize stress levels in captivity. In this study, we used meerkats (*Suricata suricatta*, Schreber 1776) as a model to examine the effect of captivity on the stress response of a highly social and cooperatively breeding species. Meerkats have been extensively studied in the wild, and the effects of natural variation in cortisol levels in response to individual and group characteristics are well documented [12–14]. Meerkats are also common in zoos, allowing the study of a relatively large sample size of varying individuals in different social and environmental conditions.

Meerkats are an obligate cooperatively breeding species of mongoose which live in groups of two to 50 animals in dry regions of southern Africa [15]. A social group consists of a dominant female and a male, which are the parents of the majority of pups born in the group [16] and both juvenile and adult subordinate helpers of both sexes, which participate in cooperative behaviours such as vigilance, babysitting and feeding the pups [16,17]. Their diet consists primarily of invertebrates and small vertebrates, which are extracted from the ground in intensive bouts of digging in sand [17]. Glucocorticoids have been linked in wild meerkats to behaviours which are important to a social species, including babysitting, pup feeding, dispersal away from the group by males and repression of reproduction in subordinate females, vigilance and response to alarm call playbacks [8,12–14,18,19].

An important consideration for the welfare of social animals is group size. Is there an optimum group size to minimize physiological stress? In the wild, meerkats which are on their own, such as evicted females or roving males, have a much higher level of fGCs than those within a group, probably because they are vulnerable to predators [12,13]. In larger groups, increasing group size brings increased protection from predators, but may also lead to increased conflict over resources and reproduction. Young [20] found fGCs to decrease with group size in relatively small (1–10, median group size = 3) dispersing coalitions of same-sex individuals, which may reflect the antipredator benefits of grouping. On the other hand, Santema [21] found that in stable groups, that also tend to be larger (2–32, median = 15), fGC concentrations increase with increasing group size, suggestive of competitive costs of large group size. These results suggest that group size may have complex relationship with measures of physiological stress, depending on the social context (dispersing versus resident groups), as well as the range of group sizes under investigation.

In this study, we investigate patterns of physiological stress in captive meerkats using non-invasive faecal sampling of 10 zoo groups. Specifically, we test: (i) whether there are consistent differences in fGC between dominant and subordinate individuals, and between sexes; (ii) how features of the captive environment, such as group size, enclosure size, season and population density, affect fGC levels; and (iii) what is the relationship between physiological stress and number of visitors. We compare the patterns of fGC in captive meerkats with those observed in the wild and discuss the factors that may affect fGC in these environments.

## 2. Material and methods

### 2.1. Faecal sample collection

We collected 140 faecal samples from meerkats living in 10 different social groups at eight zoos in England between May 2011 and January 2013 (table 1). Forty-eight of these samples, mostly of unknown origin, were collected daily from four zoo groups in summer 2011, with a further 21 samples collected from the same groups the following winter. In addition, we collected 71 samples (40 in summer 2012; 31 in winter 2012) from known individuals in six social groups using a glitter-feeding technique described in [22]. Briefly, a small quantity of food taken from the animal's daily diet was coated in very fine embossing glitter. Each piece of food was sprinkled with glitter of a particular colour, and given to a different meerkat, identified either visually or from its microchip. We observed the meerkats to check that the target individual consumed the food, and if not either removed the food item or identified the individual that ate it. Based on stress hormone releasing ACTH challenge tests and water injections carried out in individual meerkats [22], the lag time for fGC excretion as measured by our corticosterone assay (CCST) ranged between 3 and 33 h with an average (i.e. median) time lag of 22 h; faecal samples were collected during the following 36 h and the presence and colour of the glitter they contained identified on site before freezing. Time from deposition to sample collection was not recorded accurately, but it varied from a few minutes to a maximum of 3 h, with most samples being collected within half an hour from deposition. Samples were stored at −70°C for between 5 and 87 weeks before being transferred, frozen, to the Endocrinology Laboratory at the German Primate Centre in Göttingen for hormone analysis. We were not able to test for storage effects in this dataset, but it has been demonstrated that storing neat faecal samples at −20°C stabilizes faecal glucocorticoid concentrations for up to 2 years in elephants and grizzly bears [23], indicating that simple freezing reliably preserves faecal glucocorticoids long term in these and presumably also in other vertebrate species.

**Table 1.** Sample sizes from the eight zoos included in this study. (For group size, a range is shown where the number of individuals within the group varied during the study; actual group size for each sampling event is shown in figure 1.)

zoo/group	group size	no. females	no. sampled individuals	total *N* samples
Blackpool	2	1	2	5
Bristol	17	2 (7 of unknown sex)	9	13
Cotswold/1	10	4	9	15
Cotswold/2	3	0	2	4
Dartmoor	2–4	1	2	2
Longleat	14	6	7	10
Newquay	9–11	5	7	14
Paignton/1	2–4	2	5	35
Paignton/2	1–2	1	2	13
Shaldon	6–7	4	7	31
			52	140

The fGC concentrations were analysed using a corticosterone enzyme immunoassay (CCST EIA) which used the same antibody that has been used in the CCST radioimmunoassay (RIA) system validated for monitoring physiological stress responses in wild meerkats by Young *et al*. [13]. Similar to the CCST RIA, the EIA has been proved to be valid for assessing adrenocortical activity in meerkats in response to both physiological and biological stimuli [22]. Extraction was performed following previously described methodologies [24]. Samples were freeze dried at −20°C, then pulverized and sieved to remove coarse material. At this stage, sample quality was estimated by noting obvious physical qualities of the samples, such as the presence of large quantities of fur or feathers in the faeces (which was thought to result from the animals having been fed chicks the previous day), or substantial amounts of sand coating the sample, owing to the substrate from which the faeces were collected. As much extraneous sand was removed as possible. Between 0.0900 and 0.1100 g of each sample was weighed out and the weight recorded to four decimal places. Three millilitres of 80% methanol were added to each sample, then they were shaken for 10 min in a vortex and centrifuged at 3000 r.p.m. for 10 min. Two millilitres of supernatant from each sample were decanted into an Eppendorf tube and stored at −20°C until measured for glucocorticoid concentration.

Prior to assay, faecal extracts were diluted 1 : 10 (except three samples that were diluted 1 : 3 and one sample that was diluted 1 : 100) in assay buffer (0.04 M phosphate buffered saline, pH 7.2) and duplicate 50 µl aliquots were measured on microtiter plates along with 50 µl aliquots of reference standard in doubling dilutions over the range of 1.9–125 pg as described elsewhere [25]. The sensitivity of the assay was 1.9 pg. Specificity data (cross-reactivities) of the assay are reported in [25]. Intra-assay coefficients of variation for low- and high-value quality controls were 5.9% (*n* = 16) and 7.9% (*n* = 16), respectively. Respective figures for inter-assay coefficient of variation values were 8.1% (*n* = 10) and 11.4% (*n* = 10). All fGC levels reported are expressed as ng g^−1^ dry faecal mass.

### 2.2. Visitor numbers

Data on visitors were recorded on the day the samples were collected as well as the day before. The number of people within 1 m of the meerkat enclosure was counted every 2 min during each 20 min observation session, and there were on average 7.7 observation sessions (1–11) each day, amounting to 2.6 h of observations per enclosure per collection day on average. This distance was chosen to distinguish visitors that had their attention on the meerkats from visitors that were just passing by. The median and standard deviation of the number of people observed on the day were calculated, as well as on the day before, to be included as predictors in the statistical analyses (see below).

### 2.3. Statistical analysis

Data were analysed using generalized linear mixed models (GLMM) with restricted maximum likelihood estimation of random effects, as implemented in the package lme4 [26] in R v. 3.0.1 [27]. Although in most cases, all meerkats in a zoo belonged to the same group, two zoos had multiple groups of meerkats that were housed in separate enclosures (table 1). Therefore, we used meerkat group identity (ID) rather than zoo ID as a random factor in the analyses, to account for multiple sampling among group members as well as for other possible differences between groups in their genetic composition, and in their feeding and housing regimes. Individual ID was also included as a random factor, to account for multiple sampling. Sample condition as determined above (four-level factor: whether the sample contained large amounts of sand, feathers or fur, both or neither) was also included as a fixed effect in all analyses, to account for the potential effect on determination of fGCs. The fGC data were ln-transformed prior to analysis to normalize errors and meet the assumptions of parametric tests. As available data varied across samples and zoos, analyses were conducted separately for individual- and group-level factors, to maximize the sample size in each analysis as detailed below.

First, for the 59 samples for which individual identity was known, we investigated the effect of sex, dominance status (dominant or subordinate), age (in years), reproductive status of the group (pups present in the group or not) and sample condition on fGCs, including individual and group as random factors in a mixed-effects model (GLMM). Dominance was determined from zoo records of breeding patterns (only dominant meerkats breed) and confirmed by behavioural observation. A known problem in analysing fGCs is that sex differences in the absolute detected levels of fGCs can arise because of differences in the proportion of GC metabolites excreted via the faecal and urinary route, also owing to differences in the actual metabolites that are formed from cortisol/corticosterone [28–30]. We can exclude the possibility that our CCST assay picks up different metabolites in males versus females, based on an earlier study on meerkats [22], and to allow for different baseline of fGC between the sexes owing to differences in excretion routes, we included all two-way interactions between sex and the other variables in the initial analysis. To account for the possibility that status of the individual affects its response to the presence of pups, a two-way interaction between dominance and presence of pups was also included in the model.

Second, we pooled the data from 140 samples from known and unknown individuals to investigate the influence of group-level factors on fGCs. Specifically, we tested the effect of group size, the total available outdoor space (square metres), indoor space (square metres), density (group size divided by the enclosure size, in square metres), the season (summer or winter), reproductive status of the group (pups present or not) and the two-way interactions between group size and enclosure characteristics. Sample condition was controlled for and individual ID, or a running ID code for the samples of unknown origin, as well as the group ID, were included as a random effects in the analysis.

Third, the number of members of the public visiting an enclosure could directly have an effect either on the animals' glucocorticoid levels or it could be correlated with the size of the meerkat social group, and so be driving group size effects. We used the median and standard deviation of visitor numbers at the meerkat enclosure, both on the day of sample collection and the previous day, as predictors in a GLMM on 94 samples where visitor data were available. Sample quality was also controlled for, and group and individual identity fitted as random factors.

In all analyses, we first fitted all main effects and the two-way interactions were considered biologically relevant (see above). Non-significant (*p* > 0.05) interactions were removed, to allow significance testing of main terms included in the interaction, but the models were not simplified further in order to avoid problems with model selection. We report significance of terms in the main text, and the full analysis results including parameter estimates for all terms included in the models in tables 2–4.

**Table 2.** Full results from a GLMM analysis of fGCs in individually identified meerkat faeces. (Significant terms are denoted with an asterisk. For categorical variables, the parameter estimate is given relative to the value in (parentheses). Non-significant interactions were dropped from the model to allow significance testing of main terms included in the interactions, but the model was not simplified further.)

covariate	parameter estimate ± s.e.	*χ*^2^	d.f.	*p*-value
sex : age	0.002 ± 0.167	0.003	1	0.956
sex : dominance	0.956 ± 1.804	0.595	1	0.440
sex : pups	−1.644 ± 1.041	3.019	1	0.082
sex : sample condition (both)	(0)	5.402	3	0.145
dominance : pups	0.187 ± 0.989	0.100	1	0.751
sex (M)	0.456 ± 0.441	1.510	1	0.219
pups (yes)	−0.224 ± 0.600	0.076	1	0.783
age	−0.040 ± 0.152	0.177	1	0.673
dominance	−0.239 ± 0.749	0.263	1	0.608
sample condition (both)	(0)	8.331	3	0.040*
fur/feathers	−0.439 ± 1.169			
sand	1.144 ± 1.053			
neither	1.499 ± 0.945			

random effects	variance	s.d.	*N*	
group	0.450	0.671	31	
individual	0.098	0.312	10	10
residual	1.654	1.286	59	59

**Table 3.** Full results from a GLMM analysis of fGCs in all samples collected from both known and unknown individuals, in relation to group-level variables. (Significant terms are denoted with an asterisk. For categorical variables, the parameter estimate is given relative to the value in (parentheses). Non-significant interactions were dropped from the model to allow significance testing of main terms included in the interactions, but the model was not simplified further.)

covariate	parameter estimate ± s.e.	*χ*^2^	d.f.	*p*-value
group size : outdoor space	0.100 ± 0.085	0.100	1	0.340
group size : indoor space	−0.089 ± 0.186	0.723	1	0.395
group size : density	0.170 ± 2.678	0.203	1	0.653
group size	−0.056 ± 0.027	4.683	1	0.030*
outdoor space	−0.304 ± 0.118	6.706	1	0.010*
indoor space	−0.502 ± 0.112	16.60	1	<0.001*
density	0.001 ± 0.001	1.011	1	0.315
season (winter)	−0.079 ± 0.211	0.167	1	0.682
pups (yes)	−0.095 ± 0.222	0.149		0.699
condition of sample (both)	(0)	7.752	3	0.051*
feathers/fur	−0.465 ± 0.694			
sand	0.349 ± 0.692			
none	0.495 ± 0.663			

random effects	variance	s.d.	*N* levels	
ID	0.107	0.328	52	
group	0.000	0.000	10	
residual	1.040	1.020	140

**Table 4.** Full results of a GLMM analysis of fGCs in all samples collected from both known and unknown individuals, in relation to visitor numbers on previous and day of the sample collection. (Significant terms are denoted with an asterisk. For categorical variables, the parameter estimate is given relative to the value in (parentheses). Non-significant interactions were dropped from the model to allow significance testing of main terms included in the interactions, but the model was not simplified further.)

factor/covariate	parameter estimate ± s.e.	χ^2^	d.f.	*p*-value
group size : median visitors yesterday	0.028 ± 0.021	2.050	1	0.152
group size : s.d. visitors yesterday	−0.042 ± 0.033	1.734	1	0.187
group size	−0.101 ± 0.049	7.312	1	0.007*
median visitors yesterday	0.215 ± 0.078	11.04	1	<0.001*
s.d. visitors yesterday	−0.343 ± 0.106	12.12	1	<0.001*
median visitors today	−0.027 ± 0.067	0.046	1	0.830
s.d. visitors today	0.069 ± 0.045	2.651	1	0.103
condition of sample (both)	(0)	0.237	3	0.971
fur/feathers	0.059 ± 0.758	—		
sand	0.377 ± 0.872	—		
neither	0.102 ± 0.696	—		

random effects	variance	s.d.	*N* levels	
ID	0.096	0.309	31	
group	0.182	0.427	10	
residual	1.058	1.029	94	

## 3. Results

**Figure 1.**
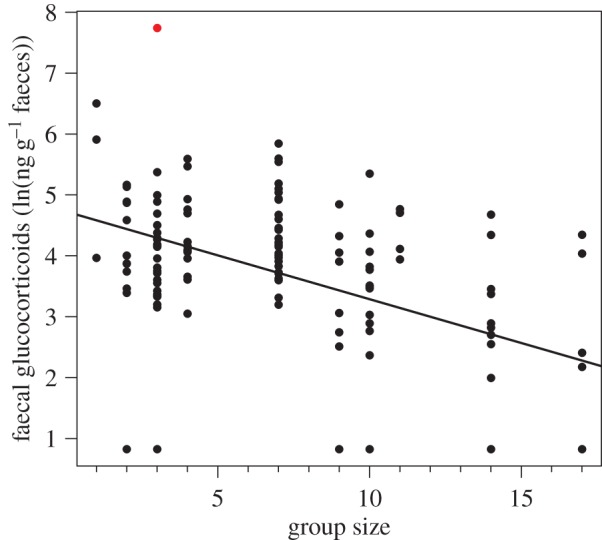
Faecal glucocorticoid levels decreased with increasing size of the social group. Dots represent ln-transformed data and the line is the model prediction after correcting for random effects of group and individual. The results were qualitatively the same when omitting the outlier (in red). Sample size: *N* = 140 samples from 52 individuals.

Seventeen of the 140 samples were found to contain glucocorticoid levels below the assay sensitivity threshold, and these were assigned the maximum level possible (the assay detection limit; 2.28 ng g^−1^ faeces for a sample weight of 0.1 g and a dilution of 3). Note that this is a very conservative approach to our data, as it leads to overestimation of levels in samples with undetectably low concentrations of fGCs, thereby potentially reducing variation in our dataset. The fGC levels in the remaining 123 samples varied between 7.34 and 2299.80 ng g^−1^ faeces, with a mean of 100.34 ng g^−1^ faeces and a median of 58.37 ng g^−1^ faeces; this difference in averages was because of a single outlier which was five times greater than the next highest value (marked in red in figure 1).

### 3.1. Do individual characteristics predict faecal glucocorticoid metabolites?

The only significant predictor of fGCs was sample condition (GLMM: $\chi_{3}^{2}=8.33$, *p* = 0.04) with samples containing neither fur or feathers, nor sand, showing the highest fGC contents (table 2). Neither the sex of the animal, its age, its dominance status, nor the two-way interactions between these variables had statistically significant effects on fGC levels.

### 3.2. Do group-level factors and characteristics of the enclosure predict faecal glucocorticoid metabolites?

Meerkats in larger groups had lower fGC levels (GLMM: *β* ± s.e. = −0.06 ± 0.03, $\chi_{1}^{2}=4.68$, *p* = 0.030; figure 1). Meerkat fGC levels also decreased with increasing size of both the indoors and outdoors enclosure (GLMM: *β* ± s.e. = −0.50 ± 0.11, $\chi_{1}^{2}=16.6$, *p* < 0.001 and −0.30 ± 0.12, $\chi_{1}^{2}=6.71$, *p* = 0.010, respectively). Again, samples with no large amounts of sand, fur nor feathers, showed highest fGC levels, and other tested factors had no effect (figure 1; table 3). Results were qualitatively the same if the outlier (marked in red in figure 1) was excluded from the analysis: group size, size of both the outdoor and indoor enclosures and sample quality were all negatively related to fGCs, while other factors had no effect (GLMM: group size: $\chi_{1}^{2}=4.29$, *p* = 0.038; outdoor space: $\chi_{1}^{2}=7.24$, *p* = 0.007; indoor space: $\chi_{1}^{2}=16.7$, *p* < 0.001; condition of sample: $\chi_{3}^{2}=8.19$, *p* = 0.042; all other *p* > 0.24).

### 3.3. What is the relationship between faecal glucocorticoid metabolite levels and visitor numbers?

**Figure 2.**
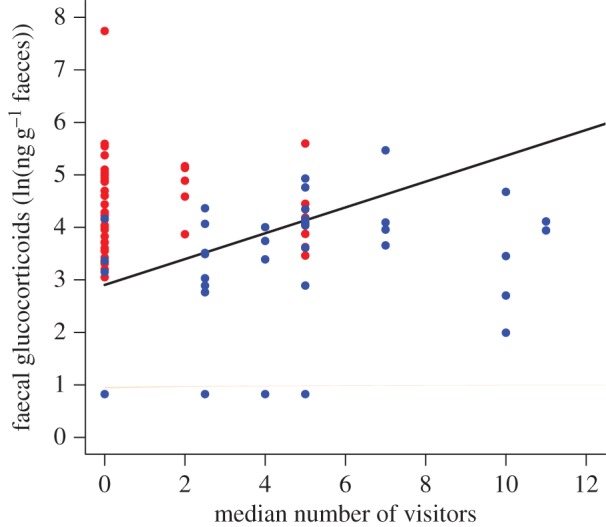
Faecal glucocorticoids increased with increasing median number of visitors at the meerkat enclosure the previous day, and levels were higher when variation in visitor numbers was lower. The line represents the model predictions for the effect of median visitor number on fGC levels, with standard deviation of the visitor number held constant (average s.d. = 4.73). For the purpose of illustration, observations with standard deviation lower than the average are marked in red, and above this are marked in blue. Sample size: *N* = 94 samples from 31 individuals.

The fGC levels increased with increasing median number of visitors observed on the previous day (*β* ± s.e. = 0.22 ± 0.08, $\chi_{1}^{2}=11.0$, *p* < 0.001), and decreased with increasing variation (s.d.) in the visitor numbers of the previous day (*β* ± s.e. = −0.34 ± 0.11, $\chi_{1}^{2}=12.1$, *p* < 0.001; figure 2). Group size was again negatively related to fGCs (*β* ± s.e. = −0.10 ± 0.05, $\chi_{1}^{2}=7.31$, *p* = 0.007). Other factors had no significant effects (table 4). Taken together, these effects show that the highest levels of physiological stress were measured in animals that were exposed to consistently high visitor numbers on the previous day, and lowest levels in animals with low median numbers of visitors. Visitor numbers on the day on which the sample was collected had no effect on fGC levels (table 4).

## 4. Discussion

We found that three main factors predicted levels of faecal glucocorticoids in zoo meerkats: the size of their social group, size of the enclosure and the number of visitors that the animals were exposed to, on the day before the sample collection. Levels of fGCs were higher in smaller groups, in groups with smaller enclosures and in groups with consistently high median number of visitors. For those samples where we could match faecal samples to specific individuals, we found no effect of age, sex or dominance class on levels of fGC, which contrasts with studies done in the wild, but matches the findings of Braga Goncalves *et al*. [22] for a captive population.

The observed negative relationship between group size and physiological stress in this study matches the findings of Young [20] working on dispersing coalitions of wild meerkats, but is contrary to the pattern found in stable, mixed sex groups by Santema [21]. In the wild, there is likely to be an optimum group size that minimizes physiological stress, as large groups are likely to experience higher within-group competition for food, whereas smaller groups face increased predation pressure [21]. Indeed, the median group size in zoos included in this study was 7 (s.d. = 4.5), which would be exceptionally small for wild meerkats in stable groups, and much closer to that seen in the dispersing coalitions in the wild. In captivity, group size has little correlation with food provision, as larger groups are fed proportionally more food, often by scatter-feeding, which reduces the ability of dominant animals to monopolize a food source [31]. This supports the idea that food limitation is likely to play a larger role in determining the levels of fGCs in the wild, whereas its role in determining variation in stress levels in captivity is negligible.

Individuals living in smaller groups in the wild may exhibit greater physiological stress because they are forced into less productive areas or subjected to greater predation, lower food intake or a trade-off between vigilance and foraging [32–34]. In captivity, many of these factors are not present, but the same pattern still emerges. It may be that, while in zoos these actual threats are not present, there is an innate hormonal stress response to being in a small group, which prepares individuals to counter these potential risks. Since in captivity food provision is generally as high or higher per animal in large groups, both lower throughput and food stress can be ruled out as causes of the group size effect in captive meerkats [31,35]. The perceived threat of attack from either conspecifics or predators, however, may still affect zoo animals. In wild meerkats, high blood cortisol levels have been linked to an increasing likelihood of performing sentry duty [36]. If a fear of attack is greater when in a small group, it would be expected that each animal should perform sentry duty more often, and that is what is observed [37]. This suggests that potential lack of control associated with uncertainty about the risk of predation and/or attack from other meerkats may be a driving force for the higher fGC observed in small groups both in captivity and in the wild.

Unsurprisingly, meerkats in larger enclosures had lower levels of fGCs, irrespective of the relative density of animals in the enclosure. In the wild, meerkat groups defend territories which can be up to several square kilometres in size, whereas enclosure sizes in zoos included in this study ranged from 34 to 300 m^2^. Meerkats need a large territory in the wild to secure sufficient food for the group, need for which is reduced under captive feeding regimes. Nevertheless, additional enclosure space may facilitate natural foraging behaviours that reduce stress, and it may also help individuals avoid or alleviate conflict, for example, by allowing subordinate individuals to physically escape aggression from dominants [13]. Other aspects of the housing environment, such as habitat enrichment or the animals' ability to hide, often correlate with enclosure size, and experimental approach would be needed in order to conclude whether these, rather than the additional space *per se*, account for the lower physiological stress levels of meerkats in larger enclosures. Complex and interacting effects of housing conditions in captivity have been found in other species. For instance, pileated Gibbons (*Hylobates pileatus*) kept in larger enclosures and less exposed to visitors had lower levels of fGCs [38], whereas captive orangutans in groups that followed a natural fission–fusion dynamic were less affected by increases in visitor numbers, than animals kept in unnaturally large, stable groups [39].

Visitor numbers also predicted fGCs in meerkats, and the median number of visitors and the standard deviation had opposite effects. The lowest fGC levels were seen in animals that had been exposed to a low number of visitors, while the highest fGC occurred when meerkats had consistently high numbers of visitors throughout the day. It is not surprising that the presence of fewer people most of the time results in a lower glucocorticoid level, as a stressful effect of visitors is seen in other species [5,39,40]. However, this does contradict a previous finding [37] that zoo meerkats exhibit lower levels of vigilance behaviour when there are more people present. As the highest peaks in visitor numbers often co-occur with feeding, this could lessen the impact on meerkats by drawing the attention of the animals away from the crowd at the enclosure. Unfortunately, data on behaviour of the meerkats were not available for this study, so we are unable to confirm whether changes in visitor numbers were associated with behavioural changes. However, our results suggest that experiencing high constant numbers of visitors would be most stressful to meerkats, whereas occasional peaks may matter less; detailed investigation of behavioural patterns associated with these changes would be useful in order to determine causality and to draw inferences on how to best minimize the impact on meerkat welfare.

In conclusion, the size of social group, size of the enclosure and the presence of visitors appear to be the most important factors in determining the physiological stress levels in captive meerkats. Meerkats in large groups had lower levels of fGCs probably owing to a higher level of perceived predation and inter-group conflict risk inherent to small groups in the wild. Unlike in studies done on wild meerkats, the age, sex and dominance status of animals did not predict fGC concentrations, which may reflect differences in determinants of physiological stress in captive versus wild animals. In captivity, individuals are likely to experience stable nutritional status irrespective of their reproductive or dominance status, which is unlikely to be true in the wild, where animals particularly in larger groups face intense competition over food. The results reported here suggest that the meerkats most at risk of unusually high and potentially detrimental levels of stress hormones are those kept in small social groups and small enclosures, with constantly high median numbers of visitors. From a husbandry policy viewpoint, although it is often not possible to control the number of visitors, zoos should be aiming to keep meerkats in larger groups and enclosures if they intend to minimize levels of physiological stress.

## Data accessibility

Supporting data are available on Figshare, doi:10.6084/m9.figshare.4665094.
